# An MRM-Based Multiplexed Quantification Assay for Human Adipokines and Apolipoproteins

**DOI:** 10.3390/molecules25040775

**Published:** 2020-02-11

**Authors:** Laura Krieg, Alexandra Schaffert, Matthias Kern, Kathrin Landgraf, Martin Wabitsch, Annette G. Beck-Sickinger, Antje Körner, Matthias Blüher, Martin von Bergen, Kristin Schubert

**Affiliations:** 1 Department of Molecular Systems Biology, UFZ, Helmholtz-Centre for Environmental Research, Permoserstraße 15, 04318 Leipzig, Germany; laura.krieg@ufz.de (L.K.);; 2 Department of Medicine, University of Leipzig, Liebigstraße 27b, 04103 Leipzig, Germany; 3 Center for Pediatric Research, Hospital for Children & Adolescents, University of Leipzig, Liebigstraße 20a, 04103 Leipzig, Germany; 4 Division of Pediatric Endocrinology Diabetes, Ulm University Medical Center, Eythstraße 24 89075 Ulm, Germany; 5 Institute of Biochemistry, University of Leipzig, Brüderstraße 34, 04103 Leipzig, Germany

**Keywords:** adipokines, apolipoproteins, multiple reaction monitoring, targeted LC-MS, obesity, human serum, SGBS cells, cell culture supernatant

## Abstract

Adipokines and apolipoproteins are key regulators and potential biomarkers in obesity and associated diseases and their quantitative assessment is crucial for functional analyses to understand disease mechanisms. Compared to routinely used ELISAs, multiple reaction monitoring (MRM)-based mass spectrometry allows multiplexing and detection of proteins for which antibodies are not available. Thus, we established an MRM method to quantify 9 adipokines and 10 apolipoproteins in human serum. We optimized sample preparation by depleting the two most abundant serum proteins for improved detectability of low abundant proteins. Intra-day and inter-day imprecision were below 16.5%, demonstrating a high accuracy. In 50 serum samples from participants with either normal weight or obesity, we quantified 8 adipokines and 10 apolipoproteins. Significantly different abundances were observed for five adipokines (adipsin, adiponectin, chemerin, leptin, vaspin) and four apolipoproteins (apo-B100/-C2/-C4/-D) between the body mass index (BMI) groups. Additionally, we applied our MRM assay to serum samples from normal weight children and human adipocyte cell culture supernatants to proof the feasibility for large cohort studies and distinct biological matrices. In summary, this multiplexed assay facilitated the investigation of relationships between adipokines or apolipoproteins and phenotypes or clinical parameters in large cohorts, which may contribute to disease prediction approaches in the future.

## 1. Introduction

Globally, the percentage of overweight and obese people has significantly increased over the last decades, reaching epidemic dimensions [1,2]. The World Health Organization (WHO) classifies the nutritional status of adults by the BMI (body mass index) with overweight defined as BMI of 25–29.9 kg/m^2^ and obesity as BMI ≥ 30 kg/m^2^ [3]. Obesity is characterized by the massive accumulation of white adipose tissue [4,5]. In previous decades, adipose tissue was believed to be only responsible for energy storage [5,6]. However, in the past 20 years it has been recognized as an endocrine organ, playing an active and important role in the whole body homeostasis by secreting a variety of different bioactive compounds, for instance, hormones and cytokines, so-called adipokines [5,6,7,8]. The discovery of leptin revealed that adipose tissue signals its energy status to the brain. Leptin regulates appetite and satiety but also energy expenditure and is directly proportional to the amount of adipose tissue [1,6]. Furthermore, adipokines have an influence on glucose metabolism (adipsin, adiponectin, asprosin, leptin, vaspin), insulin sensitivity (adiponectin, chemerin, leptin, plasminogen activator inhibitor 1 (PAI-1), retinol binding protein 4 (RBP-4), vaspin), immune responses (adipsin), inflammation (chemerin, progranulin), cell adhesion (PAI-1), and adipogenesis (chemerin) [1,6,9,10,11,12,13,14,15]. 

In obesity, the gain of adipose tissue leads to an alteration in adipokine expression and secretion, correlating with obesity-associated comorbidities, for example, type 2 diabetes, hypertension, non-alcoholic fatty liver disease, and cardiovascular diseases, which present a major health problem all over the world [1,2,3,6,16,17]. 

For certain obesity-related comorbidities such as cardiovascular diseases, altered lipoprotein serum concentrations such as LDL (low density lipoprotein)-cholesterol represent a major risk factor. Lipoproteins are particles composed of triglycerides, cholesterol (free and esterified), phospholipids, and apolipoproteins. Several epidemiological and clinical studies suggest that apolipoproteins and their ratios to one another may be a more valuable tool for the assessment of coronary heart diseases than conventional diagnostic lipoprotein assays [1,18,19]. Apolipoprotein-B100 (apo-B100) is the main structural protein of atherogenic particles (LDL), and apolipoprotein-A1 accounts for 70% of HDL (high density lipoprotein), which is considered an atheroprotective particle [20]. Therefore, the quantification of apo-B100 and apo-A1 can be used to estimate the number of LDL particles and HDL particles, respectively. However, for lipid metabolism as well as pro- and antiatherogenic pathways, other apolipoproteins (apo-A2, -A4, -C1, -C2, -C3, -C4, -D, -E) are of high importance [18]. 

Accordingly, adipokines and apolipoproteins are suitable for defining the individual obese- or lipid-metabolism-related risks for cardio metabolic diseases [21,22]. By linking a group or single adipokines and apolipoproteins to a certain obesity-associated disease or phenotype, we can get an improved understanding of the mechanisms underlying obesity. Monitoring their secretion profile over time will give insights into the efficiency of different treatment strategies [22]. 

Adipokines in human serum are found over a wide dynamic range, from pg/mL (e.g., chemerin) to µg/mL (e.g., RBP-4) [23,24,25]. Importantly, the reliable identification and quantification of proteins at low concentration ranges, especially in the context of the complexity of human serum, is very challenging [26]. Historically, enzyme-linked immunosorbent assay (ELISA) kits are routinely used for the quantification of adipokines, as they use specific and sensitive antibodies and thus overcome all the previously described challenges [27,28,29,30]. Nevertheless, the major drawback of ELISA kits is their requirement of highly specific antibodies, which are not always available for every protein of interest. Secondly, ELISAs are only to some degree multiplexable. Typically, for each protein, an ELISA has to be performed separately. This is very time-consuming and not always suitable for large cohort studies or monitoring of treatments. 

In contrast to antibody-based assays, multiple reaction monitoring (MRM) mass spectrometry (MS) allows for the simultaneous detection and quantification of several biomarkers in a single analysis run, without the need for specific antibodies. Furthermore, MRM-based assays for absolute quantification are more time- and cost-efficient per parameter and sample than ELISAs, and novel analytes can be easily added. MRM assays are based on peptides of target proteins that are unique to the proteolytically cleaved proteins [31]. The detection and quantification are based on the selection of those specific peptides and their fragments and the discrimination of all other analytes in the sample. Thus, MRM facilitates the quantification of proteins over a wide dynamic range in a complex sample matrix such as human serum. Over the last decade, peptide-based MRM assays have been successfully applied for the detection and quantitation of biomarkers, for instance, for coronary artery diseases, different types of cancer, hypertension, arthritis, and intrauterine growth restriction [26,32,33,34,35,36,37,38,39,40,41,42,43]. 

In this work, we established a multiplexed MRM-based MS assay for the quantification of circulating adipokines and apolipoproteins in different biological matrices. An optimized method was used for the quantification of 9 different adipokines and 10 apolipoproteins in serum samples of 2 human cohorts, as well as in cell culture supernatants of human adipocytes.

## 2. Results

### 2.1. Sample Preparation and Peptide Selection

**Figure 1:**
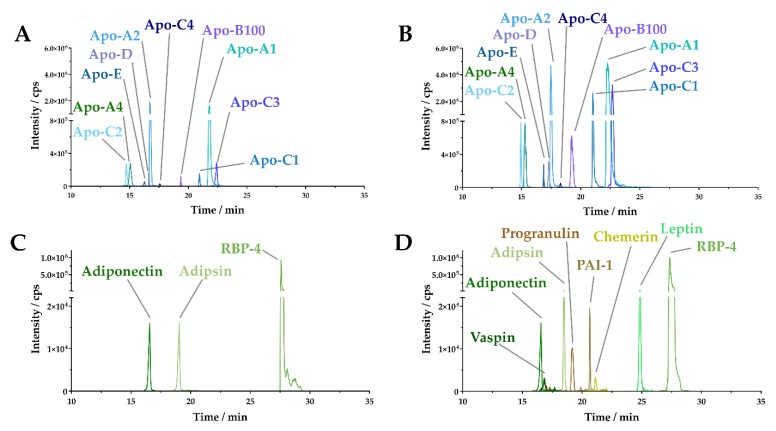
Extracted ion chromatograms (XIC) of apolipoproteins and adipokines in human serum. (**A**) XIC of 10 apolipoproteins detected in undepleted serum. (**B**) XIC of 10 apolipoproteins detected in human serum albumin (HSA)/immunoglobulin G (IgG)-depleted serum. (**C**) XIC of three adipokines detected in undepleted serum. (**D**) XIC of eight adipokines detected in HSA/IgG-depleted serum.

Previously, a reliable MRM-based workflow for the quantification of the adipokine adiponectin and RBP-4 was established. In the present study, we aimed at extending this method to 7 relevant additional adipokines as well as 10 apolipoproteins, known to play a role in obesity and obesity-related comorbidities [22]. Developing an MRM assay for the quantification of adipokines and apolipoproteins involved the in silico generation of potential candidate peptides for each targeted protein using Skyline 3.0 (see the Materials and Methods section). An array of potential candidate peptides for the apolipoproteins and adipokines (Table 1) was tested on a pooled human serum sample from five different donors. We successfully detected apo-A1, apo-A2, apo-A4, apo-B100, apo-C1, apo-C2, apo-C3, apo-C4, apo-D, and apo-E (Figure 1A). However, only three (adiponectin, adipsin, RBP-4) of nine tested adipokines could be detected (Figure 1C). In contrast to apolipoproteins, adipokines (Table 1) are of very low abundance in human serum, rendering the selection of a final candidate peptide based on only serum samples as challenging. Hence, we used recombinant human proteins, which were spiked into pooled human serum samples. 

In order to detect the low abundance adipokines in serum, we additionally implemented a serum depletion step. The depletion of human serum albumin (HSA) and immunoglobulin G (IgG) resulted in an indirect enrichment of adipokines and consequently in the successful detection of the adipokines adiponectin, adipsin, chemerin, leptin, PAI-1, progranulin, RBP-4, and vaspin (Figure 1D). In conclusion, depletion of HSA and IgG was crucial for the detection of adipokines in serum samples and did not hinder the detection of apolipoproteins (Figure 1B).

### 2.2. Assay Development

#### 2.2.1. Quantification Characteristics and Reproducibility of the MRM Method

For quantification of each targeted protein, a peptide standard was established using corresponding isotope-labeled peptides of the final candidate peptides (Table S1). To generate the calibration curve, a mixture of all isotope-labeled candidate peptides was prepared and serially diluted in HSA/IgG-depleted serum (Figures S1 and S2), followed by the determination of the limits of detection (LOD) and the lower limits of quantification (LLOQ) (Table 1). The LOD was defined as a signal-to-noise ratio of 3:1, whereas LLOQ was defined as three times the LOD. In summary, we had LODs in the range of 13.21–1025.93 ng/mL and LLOQs of 39.62–3077.80 ng/mL for all targeted proteins. 

**Figure 2:**
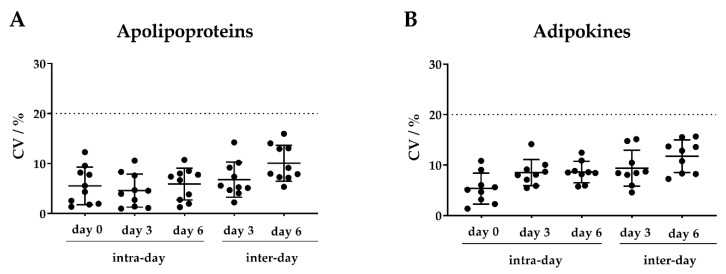
Intra- and inter-day precision of the (**A**) apolipoprotein and (**B**) adipokine multiple reaction monitoring (MRM)-based assay. One pooled isotope-labeled peptide standard mixture of all targeted apolipoproteins and adipokines was divided into three aliquots, respectively. One aliquot was directly injected into the mass spectrometer (MS) (day 0) to evaluate the intra-day precision. The other two aliquots were stored at −80 °C and subjected to the MS at day 3 and day 6, respectively. Each analysis was performed in technical quintuplicates. The coefficient of variation (CV) values are displayed in percentages for all targeted 10 apolipoproteins and 9 adipokines. Scatter plots show mean ± SD (standard deviation).

To evaluate the intra-day and inter-day accuracy, we prepared pooled samples of the 19 isotope-labeled peptide standards (Table 1). For both adipokines and apolipoproteins, we observed coefficients of variation (CVs) < 16.5% for the intra-day and inter-day assays, indicating high precision of this MRM assay (Figure 2). 

#### 2.2.2. Application of the MRM Assay: Analysis of Human Serum from Adults

**Figure 3:**
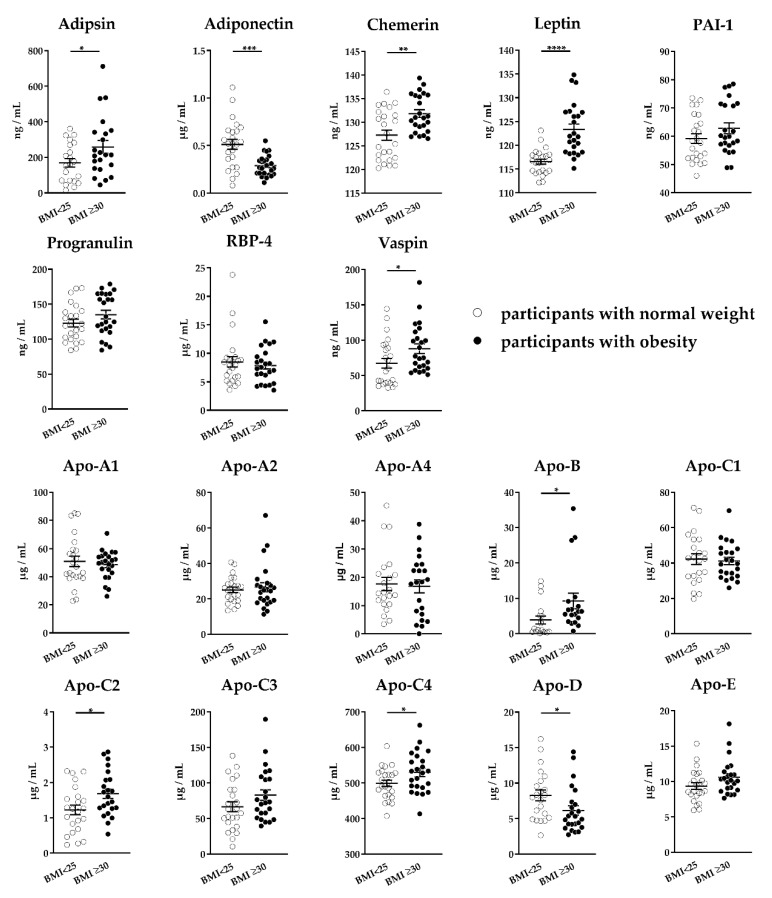
Adipokine and apolipoprotein levels in serum. Serum samples of normal weight controls (*n* = 25, body mass index (BMI) < 25) and obese patients (*n* = 25, BMI ≥ 30) were depleted, digested, and analyzed using our multiple reaction monitoring (MRM)-based assay. Scatter plots show mean ± SEM (standard error of the mean). Statistical significances were evaluated by Student’s *t*-test. * indicates statistically significant differences vs. normal weight controls: * *p*-value < 0.05, ** *p*-value < 0.01, *** *p*-value < 0.001, **** *p*-value < 0.0001.

In order to evaluate the applicability of this MRM assay, we analyzed an independent sample set consisting of serum samples from 25 normal weight and 25 obese individuals. Overall, we detected 8 adipokines and all 10 targeted apolipoproteins (Figure 3). The concentration of detected proteins ranged from 59 ng/mL (PAI-1) to 66.57 µg/mL (for apo-C3). Seven proteins (adipsin, chemerin, leptin, vaspin, apo-B100, apo-C2, apo-C4) showed significantly increased abundance in participants with obesity compared to the normal weight control group, whereas adiponectin and apo-D levels were significantly reduced. 

#### 2.2.3. Validation of the MRM Method

**Figure 4:**
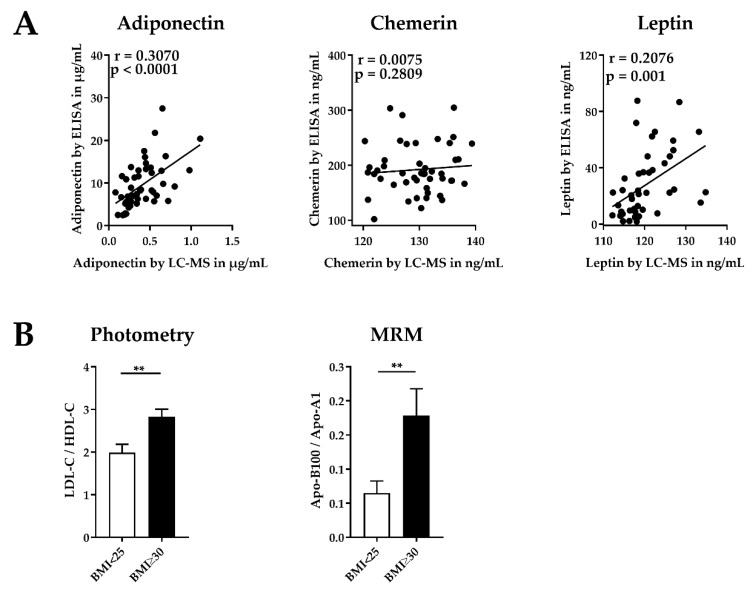
Validation of the multiple reaction monitoring (MRM)-based assay. (**A**) Correlation of adiponectin, chemerin, and leptin concentrations determined by MRM-based assay and enzyme-linked immunosorbent assay (ELISA). Data show Pearson correlation coefficients. Statistical significance was evaluated by two-tailed *t*-test. (**B**) Ratio of low density lipoprotein (LDL) and high density lipoprotein (HDL)-associated cholesterol (LDL-C/HDL-C) determined by a photometric assay (COBAS) and apolipoproteins (apo-B100/apo-A1) determined by MRM. These are indicators for the cardiovascular risk in normal weight and obese individuals. Bar graph show mean ± SEM (standard error of the mean). Statistical significances were evaluated by Student’s *t*-test. * indicates statistically significant differences vs. the normal weight control group: ** *p*-value < 0.01.

Next, we determined serum concentrations of the three adipokines adiponectin, chemerin, and leptin by ELISA for method validation and compared the results to the results obtained with our MRM assay (Figure 4A). The comparison showed significant correlations for adiponectin (*p*-value < 0.0001) and leptin (*p*-value = 0.001). No significant correlation between ELISA and MRM results was observed for chemerin. To evaluate the MRM apolipoprotein levels, we compared the apo-B100/apo-A1 ratio of our MRM method with the LDL-cholesterol/HDL-cholesterol (LDL-C/HDL-C) ratio obtained by a photometric assay (COBAS) used in clinical routine diagnostics. The results show that these ratios allow for a clear separation of obese individuals and normal weight controls (Figure 4B).

### 2.3. Further Application of the MRM Method

#### 2.3.1. Analysis of Human Serum Samples from Children

**Figure 5:**
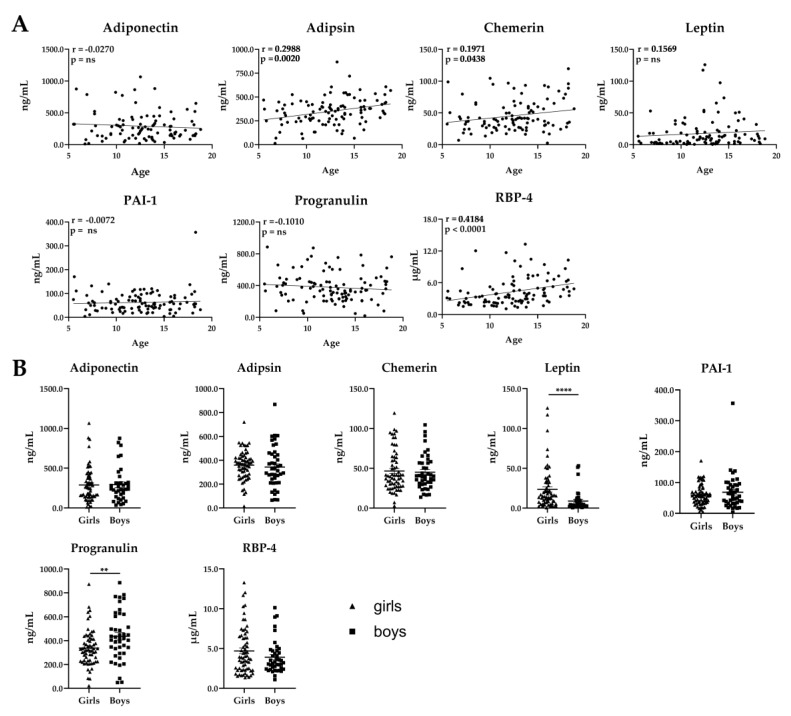
Adipokine levels in serum from normal weight children (body mass index (BMI)-standard deviation score (SDS) < 1.28). (**A**) Correlation of adipokine concentrations with age (*n* = 108, age 6–18). Data show Pearson or Spearman correlation coefficients. Statistical significance was evaluated by two-tailed *t*-test. ns, not significant. (**B**) Correlation of adipokine concentrations with sex (64 girls, 44 boys). Scatter plots show mean ± SEM (standard error of the mean). Statistical significance was evaluated by Student’s *t*-test. * indicates statistically significant differences vs. girls: ** *p*-value < 0.01, **** *p*-value < 0.0001.

**Figure 6:**
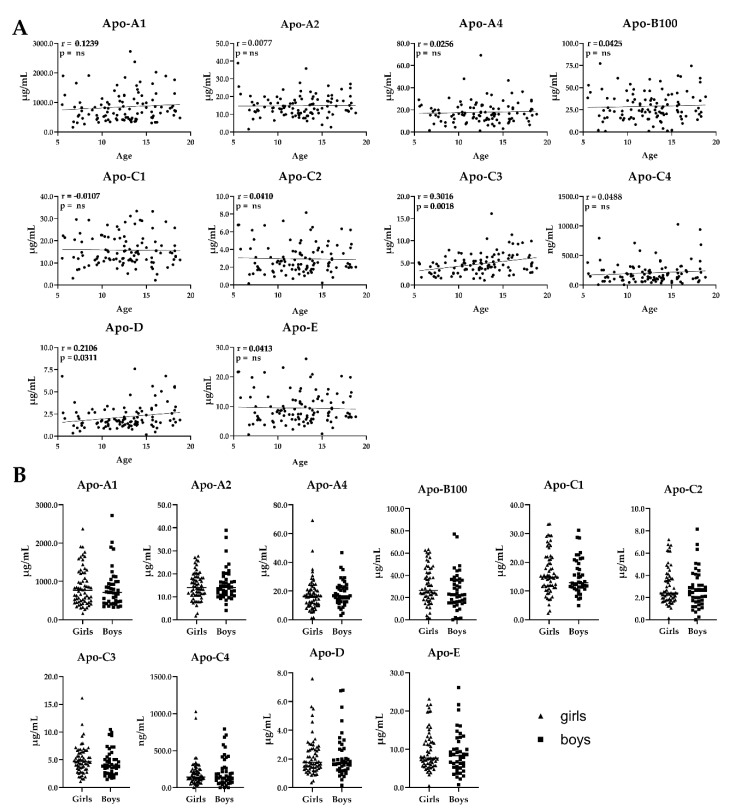
Apolipoprotein levels in serum from normal weight children (body mass index (BMI)-standard deviation score (SDS) < 1.28). (**A**) Correlation of apolipoprotein concentrations with age (*n* = 108, age 5–18). Data show Pearson or Spearman correlation coefficients. Statistical significance was evaluated by two-tailed *t*-test. ns, not significant. (**B**) Correlation of apolipoprotein concentrations with sex (64 girls, 44 boys). Scatter plots show mean ± SEM (standard error of the mean). Statistical significance was evaluated by Student’s *t*-test.

In order to evaluate the applicability of this MRM assay for large cohort studies, we analyzed an independent serum sample set obtained from 108 normal weight, BMI-matched children with our MRM assay. We quantified 7 adipokines and 10 apolipoproteins. The concentrations of the detected proteins ranged from 0.5 ng/mL (leptin) to 2725.1 µg/mL (apo-A1). Adipokine and apolipoprotein concentrations were correlated with age and sex of the children (Figure 5 and Figure 6). Three of the seven adipokines—adipsin, chemerin, and RBP-4—showed a positive correlation of serum concentrations with age. A significant difference of adipokine concentrations between girls and boys was observed for leptin and progranulin, which were decreased and increased in boys, respectively. 

Furthermore, two of the apolipoproteins (apo-C3 and apo-D) showed a positive correlation with age (apo-C3: *p* = 0.0018; apo-D: *p* = 0.0311), but there were no alterations of apolipoprotein levels between girls and boys.

#### 2.3.2. Analysis of Cell Culture Supernatants from Mature Human Adipocytes

**Figure 7:**
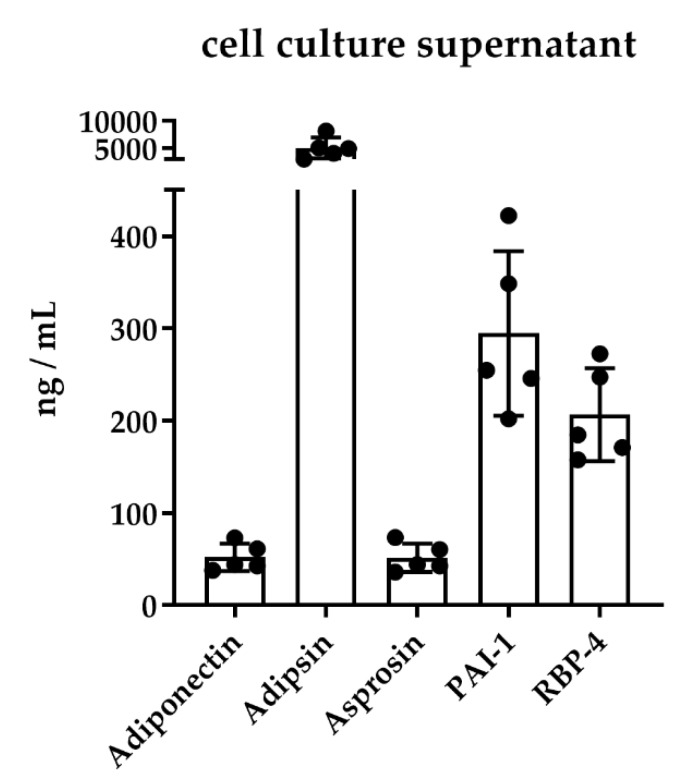
Adipokine concentrations in the cell culture supernatants of differentiated Simpson-Golabi-Behmel syndrome (SGBS) cells. SGBS cells were differentiated into mature adipocytes for 12 days and the supernatants collected at day 12 (*n* = 5) were analyzed with our multiple reaction monitoring (MRM)-based assay. Scatter plots with bars show mean ± SD (standard deviation).

To proof the feasibility of our MRM assay on different biological matrices, we applied our MRM assay to human adipocytes differentiated from Simpson-Golabi-Behmel syndrome (SGBS)preadipocytes, which are a well-established model of studying adipogenesis [44]. We detected and quantified five adipokines at day 12 of differentiation (Figure 7). Adipsin, adiponectin, PAI-1, and RBP-4 are well known as being involved in adipogenesis [23,45,46,47]. Additionally, we detected increased levels of asprosin. To our knowledge, this is the first quantification of asprosin in human adipocytes.

## 3. Discussion 

We developed a multiplexed MRM assay for the identification and absolute quantification of 9 adipokines and 10 apolipoproteins, which can either be linked to obesity-related comorbidities or are important parameters for cardiovascular risk assessment. In contrast to ELISAs, which are typically used to quantify adipokines, MRM methods do not rely on specific antibodies, require less sample amounts, and can be multiplexed. Thus, novel biomarkers of interest can be easily added. This multiplexing opportunity allows for the quantification of up to a few hundred analytes in a single run [22,31,48,49,50], whereas ELISA is limited to one biomarker that can be analyzed at one time. Albeit, it is possible to develop multiplexed ELISAs, that is, multiplexed bead array assays (MBAA), but their development is very expensive, time-consuming, and complex [51], and again requires specific antibodies. 

In this study, the levels of analyzed biomarkers varied over a wide dynamic range (five orders of magnitude). Hence, to detect also lower abundant proteins, either high abundant proteins needed to be removed or targeted proteins enriched. In the present study, we demonstrated that by depleting serum from the two most abundant proteins, HSA and IgG, five additional adipokines were quantified that were not detected without depletion. There are several depletion kits available, targeting further high abundant proteins. These might be a starting point for also identifying other low abundance adipokines of interest (e.g., protein delta homolog 1 (DLK-1), interleukin 6 (IL-6), IL-10, resistin, tumor necrosis factor (TNF), visfatin; Tables S1 and S2). 

An external calibration curve was used for quantification because it helps control for liquid chromatography (LC)-MS/MS analytical variability [20]. Potential sample matrix effects were controlled by generating the calibration curves in proteolytically cleaved HSA/IgG-depleted human serum. The intra-day CVs and inter-day CVs of our MRM assay were below 14.2% and 16.5% for adipokines and below 12.3% and 16.0% for apolipoproteins, respectively. This is in accordance with previously reported CVs for MRM-based assays and proves the high accuracy of our MRM assay [20,21].

In this study, we quantified 8 adipokines and 10 apolipoproteins in human serum. To validate our results, we compared them with concentrations obtained from ELISA kits for selected parameters. The method comparison of ELISA and MRM adipokines (adiponectin, chemerin, leptin) revealed significant correlations for adiponectin and leptin. Adipokine concentrations in both assays were comparable in terms of the observed orders of magnitude (µg/mL for adiponectin, ng/mL for chemerin and leptin). Importantly, ELISA levels showed larger variance. In contrast, the comparison of ELISA and MRM levels of chemerin showed no significant correlation, which may be explained by the existence of different chemerin isoforms [52]. For our MRM assay, we selected a representative candidate peptide, which is present in all known chemerin isoforms. Therefore, we could quantify the sum of all isoforms by our MRM assay. In contrast, the ELISA kit used in this study may not have detected all isoforms. Despite the described difference of determined adipokine levels in human serum by ELISA and MRM, we exhibited the same trends described in the literature [6,53]. For adipsin, PAI-1, progranulin, RBP-4, and vaspin, no validation using ELISA was performed, but the levels obtained in this study are in accordance with the literature (Table 2) [22,24,27,54,55,56,57,58,59,60,61,62,63,64,65,66,67,68,69,70,71,72,73,74,75,76,77]. Adipsin, chemerin, leptin, PAI-1, and vaspin were observed with significantly higher concentrations in obese individuals, thus matching previously reports as well [1,6,13,14,15,52,57,78,79]. In contrast, adiponectin is decreased in obese individuals [6,53]. The serum levels of the adipokines described here may be altered by physical activity or weight loss, and thus they may represent biomarkers for follow-up studies after bariatric by-pass surgery or diet interventions to predict therapeutic response [57]. Accordingly, a reliable quantification is of high importance and can be provided by our MRM assay.

In addition to adipokines, apolipoproteins are also useful biomarkers, especially for the development of cardiovascular diseases. In this work, we observed the same significant difference for these ratios in obese patients compared to the normal weight control group. In clinical laboratories, HDL and LDL particles are quantified by their cholesterol amount, whereas our MRM assay quantified HDL and LDL by the apoliproteins apo-A1 and apo-B100. Thus, occurring differences in the absolute ratios (LDL/HDL) may occur due to the different quantification methods. 

Herein, we aimed to develop a MS-based assay for the quantification of biomarkers in large cohorts and different biological sample types. Consequently, the assay was also tested on human serum obtained from children (*n* = 108) and cell culture supernatants of SGBS cells. First, the quantification of adipokines and apolipoproteins in serum from children revealed an increase of adipsin, RBP-4, apo-C3, and apo-D levels with increasing age. In girls, a higher level of leptin was observed when compared to boys, which is in accordance with the literature [85,86]. For the other proteins, no difference between girls and boys was observed. Second, the analysis of cell culture supernatants of SGBS cells enabled us to quantify an additional adipokine, asprosin, which is not detectable in human serum because its concentration is probably below the LOD. To our knowledge, this is the first time asprosin has been identified and quantified during the differentiation of human adipocytes. SGBS cells are a widely used and well-established model for studying human adipogenesis. SGBS cells are of human origin, thus allowing the comparison to humans. Furthermore, SGBS cells can be differentiated in serum-free media, which simplifies MS-based identification and quantification. Additionally, four more adipokines (adiponectin, adipsin, PAI-1, RBP-4) were quantified, all of which have been previously reported to be expressed during adipogenesis [23,45,46,47]. 

The reliable application to large cohorts and distinct biological matrices demonstrates the wide applicability of our developed LC-MS/MS assay.

Even though absolute values of adipokine levels are slightly different between MRM and ELISA, the results fit to the respective conditions of the samples and show the same direction and degree of differences between obese participants and the normal weight controls across different serum samples. Thus, this assay is suitable for adipokine and apolipoprotein profiling and can be applied to various studies, for instance, diet intervention studies or postoperative treatment. In summary, it may contribute to a better understanding of the pathogenesis of obesity and disease prediction. 

## 4. Material and Methods

### 4.1. Ethic Statement

The study was performed after approval by the local ethics committee (registration no. 031-2006, 017-12-23012012, 029–2006, and 265–08) and was carried out in accordance with the Declaration of Helsinki, the Bioethics Convention (Oviedo), and EU Directive on Clinical Trials (Directive 2001/20/EC). All patients were informed of the purpose, risks, and benefits of the study. Written informed consent was obtained from each subject. Ethical guidelines and EU legislation for privacy and confidentiality in personal data collection and processing were followed.

### 4.2. Reagents Chemicals and Serum Samples 

All reagents were ACS grade or higher. All solvents used were LC/MS grade. Acetonitrile, isopropanol, formic acid, and iodoacetamide (IAA) were obtained from Sigma-Aldrich (Munich, Germany). Ammonium bicarbonate was purchased from MP Biomedicals (OH, USA) and sodium deoxycholate from ABCR GmbH (Karlsruhe, Germany). Modified trypsin (sequencing grade) was obtained from Roche Diagnostics (Risch, Swiss). Tris-(2-carboxyethyl)-phosphin (TCEP) was ordered from Thermo Fischer Scientific (Bremen, Germany). 

Human recombinant proteins were expressed in *Escherichia coli* if not indicated differently and obtained from different companies: RBP-4 (human cells) from Thermo Fischer Scientific (Bremen, Germany); adiponectin (Hi-5 insect cells), Leptin, and Vaspin from PeproTech (Hamburg, Germany); Adipsin from Prospec (Rehovot, Israel); Progranulin (HEK 293 cells) and PAI-1 from Sigma-Aldrich (München, Germany); and chemerin was synthesized in the group of Prof. Dr. A. Beck-Sickinger (University of Leipzig, Leipzig, Germany) [87].

Isotope-labeled candidate peptides for absolute quantification were synthesized at the Interdisciplinary Centre for Clinical Research (purity > 98 %) (IZKF, Leipzig, Germany). 

Human serum samples were obtained from the outpatient’s clinic at the Department of Medicine (University of Leipzig, Leipzig, Germany). For this study, 50 individuals were selected to compare men and women with a BMI of either below 25 kg/m² or above 30 kg/m² (Table 3). The study was approved by the local ethics committee (registration no. 031-2006 and 017-12-23012012) and participants gave their written informed consent.

Additionally, 108 normal-weight, BMI-matched children were selected (Table 4). The study was approved by the local ethics committee (029–2006, 265–08) and is registered in the National Clinical Trials database (NCT01605123, NCT02208141). 

### 4.3. Human Serum from Adults and Children

#### 4.3.1. Protein Depletion

As an initial step, the two most abundant proteins (human serum albumin, IgG) were removed by HSA/IgG multiple affinity removal spin cartridges from Agilent Technologies (Santa Clara, CA, USA). The depletion procedure was performed at room temperature according to the manufacturer’s instructions. The combined flow through fractions and the bound fraction were stored at −80 °C until proteolytic cleavage. 

#### 4.3.2. Proteolytic Cleavage of Human Serum

Aliquots of recombinant human adipokines (2 µg) and depleted human serum (350 μg protein) were diluted with ammonium bicarbonate (25 mmol/L) to achieve a final concentration of 0.2 µg/µL and 100 μg/*μ*L, respectively, before sodium deoxycholate (5%, w/v) was added to denature the proteins. Proteins were reduced with TCEP (5 mmol/L) and alkylated with IAA (10 mmol/L, 37 °C, 30 min, darkness, 550 rpm). Trypsin was dissolved in ammonium bicarbonate (25 mmol/L) and added to the sample (30:1 protein to enzyme ratio, overnight, 37 °C, 550 rpm). The digestion was stopped with formic acid (0.5%, *v*/*v*), precipitated sodium deoxycholate was removed by centrifugation (5 min, 16,000 rpm), and the supernatants were stored at −20 °C.

### 4.4. Human Preadipocytes (SGBS Cells)

#### 4.4.1. Differentiation of Human Preadipocytes

SGBS cells were differentiated from preadipocytes to mature adipocytes for 12 days, as described previously [44]. Briefly, SGBS preadipocytes were cultured in Dulbecco’s Modified Eagle Medium (DMEM)/Ham’s F12 culture media containing 33 µM biotin and 17 µM pantothenic acid. Preadipocytes were grown to confluence in culture media containing 10% fetal calf serum FCS. The differentiation was conducted in culture media containing 0.13 nM apo-transferrin, 0.2 nM triiodotyronine, 20 nM insulin, and 100 nM hydrocortisone under FCS-free conditions. During the first 4 days, 2 µM rosiglitazone, 500 µM 3-isobutyl-1-methylxanthine, and 25 nM dexamethasone were added. Cell culture supernatant was collected at day 0 and day 12. The experiment was performed in quintuplicate.

#### 4.4.2. Proteolytic Cleavage of Cell Culture Supernatant

Cell culture supernatants (1.8 mL) were processed by the FASP approach (filter aided sample preparation) using 10 kDa Vivacon molecular weight cut off filters (Sartorius, Göttingen, Germany), as described previously [88]. In brief, filters were prepared using 100 µL urea buffer A (UA) (8 M urea in 0.1 M Tris-HCl, pH 8.5) and centrifuged (20 min, 14,000× *g*). Afterwards, the sample was loaded onto the filter and centrifuged (40 min, 14,000× *g*). A total of 200 µL of buffer UA was pipetted onto the filter and centrifuged, and the eluate was then discarded. Then, 100 µL of buffer UA was added and 10 µL of sample was pipetted off the filter and used for determination of protein concentrations using Pierce 660 nM Protein assay (Thermofischer). The filter was centrifuged again. The proteins were reduced using 200 µL dithiothreitol (100 mM in UA, 37 °C, 30 min) and centrifuged and alkylated with 200 µL IAA (50 mM in UA, 10 min, darkness). The filter unit was centrifuged again and washed twice with 100 µL of urea buffer B (UB) (8 M urea in 0.1 M Tris-HCl, pH 8.0). Following on from this, proteins were digested using trypsin (30:1 protein to enzyme ratio, overnight, 37 °C). The released peptides were then collected by centrifugation (60 min, 14,000× *g*) and washing of the filter unit using 50 µL NaCl (500 mM). The digestion was stopped using 10 µL formic acid (10%, *v*/*v*). Samples were desalted using solid phase extraction OASIS cartridges (Oasis HLB 1 cc, 10 mg, Waters). The cartridges were washed with 1 mL methanol and equilibrated two times with 1 mL formic acid (0.1%, *v*/*v*). Then, the peptide was loaded onto the cartridge, washed three times with formic acid (0.1%, *v*/*v*), and eluted with 500 µL acetonitrile (70%, *v*/*v*) containing formic acid (0.1%, *v*/*v*). The samples were dried under vacuum and stored at -20 °C until analysis.

### 4.5. NanoRP-UPLC-MS/MS

All samples were analyzed on a nanoAcquity ultra performance liquid chromatography (UPLC) system (Waters GmbH, Eschborn, Germany) coupled on-line to a QTRAP 5500 mass spectrometer (AB Sciex, Darmstadt, Germany) equipped with an ESI Nanospray III source (AB Sciex) using PicoTip emitters (non-coated silica tip, 10 μm, New Objective, Woburn, USA). Peptides (4 µL) were trapped on a nanoAcquity Symmetry C_18_-column (internal diameter (ID) 180 µm, length 20 mm, particle diameter 5 µm) at a flow rate of 10 µL/min (2% eluent B) for 3 min and separated on a BEH 130 column (C_18_-phase, ID 75 µm, length 150 mm, particle diameter 1.7 µm; 40 °C) using a flow rate of 350 nL/min. Eluent A was water containing formic acid (0.1%, *v*/*v*) and eluent B acetonitrile with *i*-propanol (2%, *v*/*v*) and formic acid (0.1%, *v*/*v*). Peptides were eluted by a consecutive linear gradient from 2% to 30% (28 min) and 30% to 85% (5 min) eluent B.

The QTRAP 5500 was operated using the following parameters: interface heater temperature 150 °C, ion spray voltage 2.3 kV, curtain gas 20 psi, ion source gas 115 psi. Optimized declustering potential, entrance potential, and collision cell exit potential for multiple reaction monitoring (MRM) experiments were set to 80, 10, and 10 V, respectively. Doubly charged precursor ions were selected in Q1 and monitored together with three singly charged y- or b-ions of each peptide in Q3 (Table S1). Collision energies for each transition were optimized by MRM-Pilot (AB Sciex) iteration experiments. Mass spectra were recorded in positive ion mode for a *m/z* range from 50 to 1250 in the quadrupole mass analyzer at unit resolution with a scan speed of 10 Da/s. Data were acquired and analyzed using Analyst software 1.6.2 (AB Sciex, Darmstadt, Germany). 

For the quantification of adipokines and apolipoproteins, an external calibration curve was generated using isotope-labeled peptides (one for each candidate peptide) spiked into human serum. These standard solutions ranged from 1 to 800 fmol and were additionally used for determining the limits of detection (LOD) and lower limits of quantification (LLOQ). The calibration curve was obtained by MultiQuant 3.0.3 (AB Sciex) using weighted (1/x) least-squares regression analysis.

### 4.6. Selection of Potential Candidate Peptides

For each adipokine and apolipoprotein of interest, a list of possible candidate peptides was generated by the Skyline 3.0 software (MacCoss Lab Software, Seattle, USA) software using certain criteria. The proteolytic enzyme was trypsin, cleaving after lysine (K) and arginine (R). The peptide sequence has to be unique in the human proteome, no missed cleavage sites or methionine (M) and cysteine (C) were allowed. The peptide should be at least 5 amino acids long, but not exceed 20. Only monoisotopic precursors were predicted, with a charge of +2 or +3 and fragment ion charges of +1 or +2. Only y- and b-ions were included. 

### 4.7. Data Analysis

Acquired data were viewed and analyzed in Analyst software 1.6.2, Skyline software 3.0, and MultiQuant 3.0.3. Statistical analysis was carried out in GraphPad Prism 8. Quantification data were tested for normality and statistical significances were evaluated by Student’s *t*-test or Mann–Whitney U rank sum test. For all comparisons, Pearson‘s correlation coefficient and Spearman‘s rank correlation coefficient were used for normally or non-normally distributed data, respectively.

### 4.8. Validation of MRM-Based Method Using ELISA

For comparison and validation of the MRM-based results, ELISA kits were used to determine the concentration of adiponectin (Adiponectin ELISA, Adipogen, San Diego, CA, USA), chemerin (Chemerin ELISA, Biovendor, Karasek, Czech Republic), and leptin (Leptin ELISA, Mediagnost, Reutlingen, Germany) in each serum sample, as described previously [82].

## 5. Conclusions

In the present study, we demonstrated the feasibility of a targeted LC-MS/MS-based assay for the identification and quantification of targeted peptides and their corresponding adipokines and apolipoproteins in human serum and cell culture supernatant. The multiplexicity of this assay might lead to an increase of detectable parameters in clinical research in order to gain deeper insights in the correlation of adipokines, apolipoproteins, and obesity-related comorbidities. By automation of the experimental workflow and addition of other important biomarkers, this multiplexed MRM assay may be of high interest for large cohort studies.

## Figures and Tables

**Table 1:** Calibration curve parameters, limits of detection (LOD), and lower limits of quantification (LLOQ) for the quantification of different adipokines and apolipoproteins in human serum.

Protein	External Calibration Curve			LOD		LLOQ	
	Slope	Intercept	*R^2^*	Peptide (ng/mL)	Protein (ng/mL)	Peptide (ng/mL)	Protein (ng/mL)
**Adipsin**	3.11×10^4^	1.41×10^6^	0.97	0.63	21.63	1.88	64.88
**Adiponectin**	3.22×10^4^	−7.72×10^4^	0.99	0.71	13.21	2.13	39.62
**Asprosin**	2.11×10^4^	−4.50×10^6^	0.96	6.02	85.00	18.05	255.00
**Chemerin**	9.71×10^4^	−5.97×10^5^	0.99	1.91	27.93	5.74	83.78
**Leptin**	9.71×10^4^	−5.97×10^5^	0.99	2.30	37.28	6.89	111.85
**PAI-1**	3.78×10^5^	−1.29×10^6^	0.99	0.34	22.53	1.03	67.59
**Progranulin**	7.53×10^4^	2.08×10^5^	0.99	0.50	31.77	1.51	95.32
**RBP-4**	1.27×10^5^	−2.54×10^6^	0.99	30.09	575.25	90.27	1725.75
**Vaspin**	1.20×10^5^	−9.41×10^4^	0.99	0.44	23.59	1.31	70.76
**Apo-A1**	7.32×10^3^	−6.99×10^5^	0.98	46.86	1025.93	140.57	3077.80
**Apo-A2**	5.08×10^4^	3.12×10^5^	0.99	1.58	18.63	4.74	55.88
**Apo-A4**	1.97×10^4^	3.55×10^4^	0.99	1.65	75.67	4.94	227.00
**Apo-B100**	1.62×10^4^	8.44×10^3^	0.99	1.91	859.34	5.72	2578.03
**Apo-C1**	2.69×10^3^	−4.43×10^4^	0.99	4.04	31.11	12.11	93.32
**Apo-C2**	6.02×10^4^	5.98×10^5^	0.99	1.73	18.81	5.20	56.42
**Apo-C3**	5.00×10^3^	−1.19×10^5^	0.99	20.03	180.87	60.08	542.60
**Apo-C4**	3.65×10^4^	−7.26×10^4^	0.99	1.80	24.26	5.39	72.77
**Apo-D**	4.03×10^4^	2.40×10^5^	0.99	2.06	35.46	6.18	106.38
**Apo-E**	1.30×10^4^	−1.47×10^5^	0.99	5.01	120.51	15.03	361.54

**Table 2:** Concentrations of adipokines in adult serum quantified with our MRM assay and by ELISA obtained from the literature.

Protein	MRM	Literature (ELISA)
**Adipsin**	15.2–711.4 ng/mL	467–5148 ng/mL [57,58]
**Adiponectin**	0.1–1.1 µg/mL	0.02–10.3 µg/mL [70,71,80]
**Chemerin**	120.3–139.4 ng/mL	0.6–204.8 ng/mL [24,69,81]
**Leptin**	112.2–134.84 ng/mL	1.2–101.3 ng/mL [71,72,81,82]
**PAI-1**	46.01–78.5 ng/mL	1.8–270 ng/mL [65,66,67,68]
**Progranulin**	84.1–178.9 ng/mL	5.2–386.0 ng/mL [59,74,83]
**RBP-4**	3.5–23.8 µg/mL	1.5–163 µg/mL [22,55,56,84]
**Vaspin**	32.6–181.7 ng/mL	0.3–17.9 ng/mL [61,62,63,64,82]

**Table 3:** Clinical characteristics of individuals that were assigned to the group of lean (BMI < 25 kg/m²) or obese (BMI > 30 kg/m²) individuals. Data are presented as mean ± SD.

	Normal Weight (*n* = 25)	Obese (*n* = 25)
**Sex** (w/m)	21/4	16/9
**Age** (years)	51.2 ± 18.0	54.1 ± 11.2
**Body weight** (kg)	61.3 ± 10.2	129.0 ± 21.7
**BMI** (kg/m^2^)	21.7 ± 2.5	46.6 ± 6.4
**HDL-cholesterol** (mmol/L)	1.7 ± 0.4	1.3 ± 0.3
**LDL-cholesterol** (mmol/L)	3.2 ± 0.8	3.6 ± 0.8
**Type 2 Diabetes** (yes/no)	6/19	19/6

**Table 4:** Clinical characteristics of normal weight children (BMI standard deviation score (SDS) < 1.28). Data are presented as mean ± SD.

	Girls (*n* = 64)	Boys (*n* = 44)
**Age** (years)	12.8 ± 3.6	12.2 ± 3.2
**BMI** (SDS)	−0.3 ± 0.8	−0.2 ± 0.7

## Supplementary Materials

The following are available online: Figure S1: Calibration curves (*n* = 5) of 16 adipokines. Isotope-labeled peptides were spiked in to HSA/IgG-depleted human serum and serially diluted. Figure S2: Calibration curves (*n* = 5) of 10 apolipoproteins. Isotope-labeled peptides were spiked into HSA/IgG-depleted human serum and serially diluted. Table S1: MS transitions and MRM parameters for proteolytic tryptic peptides of 16 adipokines and 10 apolipoproteins and corresponding stable isotope-labeled peptides used as standard (S). For each biomarker, the final candidate peptide amino acid sequence and *m/z* is displayed. Furthermore, the transitions used for quantification and identification (fragment ions) and optimized declustering potential (DP) and collision energy (CE) are listed for each peptide. The same information is provided for the isotope-labeled peptide standards. Table S2: Calibration curve parameters, limits of detection (LOD), and lower limits of quantification (LLOQ) for the quantification of different adipokines and apolipoproteins in human serum.
