# Supplementary material for: An MRM-Based Multiplexed Quantification Assay for Human Adipokines and Apolipoproteins
Source: Molecules. 2020 Feb 11;25(4):775. doi: 10.3390/molecules25040775 (PMC7070386; doi:10.3390/molecules25040775)
Supplement: Supplementary file 1 [file molecules-25-00775-s001.pdf]

Supplement

Table S1. MS transitions and MRM parameters for proteolytic tryptic peptides of 16 adipokines and 10 apolipoproteins and corresponding stable isotope-labeled peptides used as standard (S). For each biomarker the final candidate peptide amino acid sequence and *m/z* is displayed. Furthermore, the transitions used for quantification and identification (fragment ions) and optimized declustering potential (DP) and collision energy (CE) are listed for each peptide. The same information is provided for the isotope-labeled peptide standards.

Protein	Proteolytic peptide	*m/z*	Quantifier		Qualifier 1 and 2		DP / V	CE / V
			Fragment ion	*m/z*	Fragment ion	*m/z*		
**Adiponectin**	GDIGETGVPGAEGPR	706.34	y_7	683.35	y_9 / b_8	839.44 / 729.34	125 / 106.3 / 125	34 / 34.3 / 27
**Adipsin**	LYDVLR	389.73	y_5	665.36	y_4 / b_4	502.30 / 246.13	73	16.9
**Asprosin**	EGISYLHFTK	398.88	y_7^{+2}	448.24	y_5^{+2} / y_6^{+2}	323.19 / 404.72	66.3 / 45.3 / 67.0	15.7 / 15.0 / 14.7
**Chemerin**	GLQVALEEFHK	424.23	b_2	171.11	y_2^{+2} / y_9^{+2}	142.59 / 553.80	67.0 / 59.7 / 51.0	17.0 / 20.7 / 14.3
**DLK-1**	LPSGYGLAYR	548.79	y_9^{+2}	492.25	y_5 / y_8	579.32 / 886.44	118.0 / 89.3 / 78.7	26.3 / 31.7 / 30.7
**GLP-1**	HAEGTFTSDVSSYLEGQAAK	700.00	y_7	716.40	y_6 / y_{10}	603.31 /	73	29.7 / 26.7 / 29.7
**IL-6**	EALAENNLNLPK	663.36	y_2	244.17	y_8 / y_9	941.51 /	114.0 / 79.7 / 127	30.7 / 31.0 / 30.3
**IL-10**	DQLDNLLLK	536.31	y_7	828.52	y_5 / y_6	600.41 / 715.43	100.7 / 95.7 / 70.7	23.0 / 28.3 / 23.0
**Leptin**	DLLHVLAFSK	381.56	y_8^{+2}	457.78	y_4 / y_7^{+2}	452.25 / 401.23	61.3 / 62.7 / 70.0	13.3 / 15.3 / 18.3
**PAI-1**	FIINDWVK	517.79	y_6	774.41	y_5 / b_2	661.33 / 261.16	112.0 / 104.0 / 98.3	21.0 / 22.7 / 20.7
**Progranulin**	GSEIVAGLEK	501.78	y_7	729.45	y_5 / y_6	517.30 / 616.37	95.0 / 49.3 / 48.3	22.3 / 21.3 / 23
**RBP-4**	YWGVASFLQK	599.82	y_6	693.39	y_8 / b_2	849.48 / 350.15	83.0 / 79.0 / 91	26.3 / 27.0 / 27.0
**Resistin**	IQEVAGSLIFR	616.85	y_7	763.45	y_6 / y_9	692.41 / 991.56	106.3 / 96.7 / 112.30	30.7 / 29.7 / 28.3
**TNFa**	ANALLANGVELR	620.85	y_7	758.42	y_6 / y_8	687.38 / 871.50	60.7 / 85.3 / 96.3	29.0 / 26.7 / 31.0
**Vaspin**	VVDVSVPR	435.76	y_4	458.27	y_5 / y_6	557.34 / 672.38	59.0 / 62.7 / 53.3	18.3 / 23.3 / 20.3
**Visfatin**	SYSFDEIR	508.74	y_6	766.37	y_5 / b_2	679.34 / 251.10	95.0 / 86.3 / 68.3	20.7 / 21.3 / 20.0
**Apo-A1**	DYVSQFEGSALGK	700.84	y_{10}	1023.51	y_9 / y_{11}	936.48 /	82.2	34.1
**Apo-A2**	EQLTPLIK	471.29	y_4	470.33	y_5 / y_6	571.38 / 684.47	65.5	25.8
**Apo-A4**	LAPLAEDVR	492.28	y_5	589.29	y_6 / y_7	702.38 / 799.43	67.0	26.6
**Apo-B**	EVYGFNPEGK	570.27	y_7	748.36	y_5 / y_8	544.27 / 911.43	72.7	29.4
**Apo-C1**	EWFSETFQK	601.28	y_4	523.29	y_6 / y_7	739.36 / 886.43	75.0	30.5
**Apo-C2**	TYLPAVDEK	518.27	y_6	658.34	y_5 / y_7	561.29 / 771.42	68.9	27.5
**Apo-C3**	GWVTDGFSSLK	598.80	y_9	953.49	y_7 / y_8	753.38 / 854.43	74.8	30.4

Protein	Proteolytic peptide	*m/z*	Quantifier		Qualifier 1 and 2		DP / V	CE / V
			Fragment ion	*m/z*	Fragment ion	*m/z*		
**Apo-C4**	ELLETVVNR	532.80	y_5	588.35	y_6 / y_7	717.39 / 830.47	70.2	28.2
**Apo-D**	NILTSNNIDVK	615.84	y_7	789.41	y_8 / y_9	890.46 /	76.0	31.0
**Apo-E**	AATVGSLAGQPLQER	749.40	y_7	827.44	y_5 / y_8	642.36 / 898.47	85.7	35.8
**Apo-A1**	DYVSQFEGSALGK	700.84	y_{10}	1023.51	y_9 / y_{11}	936.48 /	82.2	34.1
**S Adiponectin**	GDIGETG**V**PGAEGPR	709.35	y_9	845.45	y_7 / b_8	683.35 / 735.36	125 / 106.3 / 125	34 / 34.3 / 27
**S Adipsin**	LYD**V**LR	392.73	y_4	508.31	y_5 / b_4	671.38/ 249.14	73	16.9
**S Asprosin**	EGISYLH**F**TK	402.22	y_7^{+2}	453.25	y_5^{+2} / y_6^{+2}	328.20 / 409.73	66.3 / 45.3 / 67.0	15.7 / 15.0 / 14.7
**S Chemerin**	GLQ**V**ALEEFHK	426.24	b_2	171.11	y_2^{+2} / y_9^{+2}	142.59 / 553.80	67.0 / 59.7 / 51.0	17.0 / 20.7 / 14.3
**S DLK-1**	LPSGYG**L**AYR	552.30	y_9^{+2}	495.76	y_5 / y_8	586.34 / 893.46	118.0 / 89.3 / 78.7	26.3 / 31.7 / 30.7
**S GLP-1**	HAEGTFTSD**V**SSYLEGQAAK	702.00	y_7	716.40	y_6 / y_{10}	603.31 /	73	29.7 / 26.7 / 29.7
**S IL-6**	EA**L**AENNLNLPK	666.87	y_2	244.17	y_8 / y_9	941.51 /	114.0 / 79.7 / 127	30.7 / 31.0 / 30.3
**S IL-10**	DQ**L**DNLLLK	539.81	y_7	835.54	y_5 / y_6	600.41 / 715.43	100.7 / 95.7 / 70.7	23.0 / 28.3 / 23.0
**S Leptin**	DLLH**V**LAFSK	383.56	y_8^{+2}	460.78	y_4 / y_7^{+2}	452.25 / 404.24	61.3 / 62.7 / 70.0	13.3 / 15.3 / 18.3
**S PAI-1**	FIINDW**V**K	520.79	y_6	780.43	y_5 / b_2	667.34 / 261.16	112.0 / 104.0 / 98.3	21.0 / 22.7 / 20.7
**S Progranulin**	GSEI**V**AGLEK	504.78	y_7	735.46	y_5 / y_6	517.30 / 622.38	95.0 / 49.3 / 48.3	22.3 / 21.3 / 23
**S RBP-4**	YWG**V**ASFLQK	602.82	y_6	693.39	y_8 / b_2	855.50 / 350.15	83.0 / 79.0 / 91	26.3 / 27.0 / 27.0
**S Resistin**	IQE**V**AGSLIFR	619.86	y_7	763.45	y_6 / y_9	692.41 / 997.57	106.3 / 96.7 / 112.30	30.7 / 29.7 / 28.3
**S TNFa**	ANALLANG**V**ELR	623.86	y_7	764.43	y_6 / y_8	693.39 / 877.51	60.7 / 85.3 / 96.3	29.0 / 26.7 / 31.0
**S Vaspin**	VVD**V**SVPR	438.76	y_4	458.27	y_5 / y_6	563.35 / 678.38	59.0 / 62.7 / 53.3	18.3 / 23.3 / 20.3
**S Visfatin**	SYSFDE**I**R	512.25	y_6	773.39	y_5 / b_2	686.36 / 251.10	95.0 / 86.3 / 68.3	20.7 / 21.3 / 20.0
**S Apo-A1**	DY**V**SQFEGSALGK	703.85	y_{10}	1023.51	y_9 / y_{11}	936.48 /	82.2	34.1
**S Apo-A2**	EQ**L**TPLIK	474.8	y_4	470.33	y_5 / y_6	571.38 /	65.5	25.8
**S Apo-A4**	LAPLAED**V**R	495.29	y_5	595.31	y_6 / y_7	708.39 / 802.45	67.0	26.6
**S Apo-B**	E**V**YGFNPEGK	573.28	y_7	748.36	y_5 / y_8	544.27 / 911.43	72.7	29.4
**S Apo-C1**	EWFSET**F**QK	606.29	y_4	533.31	y_6 / y_7	749.39 / 896.46	75.0	30.5
**S Apo-C2**	TYLPA**V**DEK	521.28	y_6	664.35	y_5 / y_7	567.30 / 777.44	68.9	27.5
**S Apo-C3**	GW**V**TDGFSSLK	601.81	y_9	959.51	y_7 / y_8	753.38 / 854.43	74.8	30.4
**S Apo-C4**	ELLET**V**VNR	539.81	y_5	594.36	y_6 / y_7	723.40 / 836.49	70.2	28.2
**S Apo-D**	NILTSNNID**V**K	618.85	y_7	795.42	y_8 / y_9	896.47 /	76.0	31.0
**S Apo-E**	AAT**V**GSLAGQPLQER	752.41	y_7	827.44	y_5 / y_8	642.36 / 898.47	85.7	35.8

Table S2. Calibration curve parameters, limits of detection (LOD) and lower limits of quantification (LLOQ) for the quantification of different adipokines and apolipoproteins in human serum.

Protein	external calibration curve			LOD		LLOQ	
	slope	intercept	R^2	peptide (ng/mL)	protein (ng/mL)	peptide (ng/mL)	protein (ng/mL)
**Adipsin**	$3.11 \cdot 10^4$	$1.41 \cdot 10^6$	0.97	0.63	21.63	1.88	64.88
**Adiponectin**	$3.22 \cdot 10^4$	$-7.72 \cdot 10^4$	0.99	0.71	13.21	2.13	39.62
**Asprosin**	$2.11 \cdot 10^4$	$-4.50 \cdot 10^6$	0.96	6.02	85.00	18.05	255.00
**Chemerin**	$9.71 \cdot 10^4$	$-5.97 \cdot 10^5$	0.99	1.91	27.93	5.74	83.78
**DLK-1**	$6.30 \cdot 10^4$	$-6.30 \cdot 10^5$	0.99	6.62	247.80	19.85	743.40
**GLP-1**	$3.35 \cdot 10^3$	$-1.49 \cdot 10^6$	0.98	105.21	208.35	315.63	625.05
**IL-6**	$1.36 \cdot 10^4$	$-1.54 \cdot 10^5$	0.99	5.33	94.87	15.98	284.62
**IL-10**	$4.20 \cdot 10^4$	$-1.46 \cdot 10^6$	0.98	10.78	205.17	32.33	615.51
**Leptin**	$9.71 \cdot 10^4$	$-5.97 \cdot 10^5$	0.99	2.30	37.28	6.89	111.85
**PAI-1**	$3.78 \cdot 10^5$	$-1.29 \cdot 10^6$	0.99	0.34	22.53	1.03	67.59
**Progranulin**	$7.53 \cdot 10^4$	$2.08 \cdot 10^5$	0.99	0.50	31.77	1.51	95.32
**RBP-4**	$1.27 \cdot 10^5$	$-2.54 \cdot 10^6$	0.99	30.09	575.25	90.27	1725.75
**Resistin**	$9.25 \cdot 10^3$	$-2.56 \cdot 10^5$	0.97	12.38	114.19	37.13	342.57
**TNFa**	$1.23 \cdot 10^5$	$-3.13 \cdot 10^6$	0.99	31.14	616.10	93.43	1848.30
**Vaspin**	$1.20 \cdot 10^5$	$-9.41 \cdot 10^4$	0.99	0.44	23.59	1.31	70.76
**Visfatin**	$1.53 \cdot 10^5$	$5.94 \cdot 10^5$	0.99	0.51	27.76	1.53	83.28
**Apo-A1**	$7.32 \cdot 10^3$	$-6.99 \cdot 10^5$	0.98	46.86	1025.93	140.57	3077.80
**Apo-A2**	$5.08 \cdot 10^4$	$3.12 \cdot 10^5$	0.99	1.58	18.63	4.74	55.88
**Apo-A4**	$1.97 \cdot 10^4$	$3.55 \cdot 10^4$	0.99	1.65	75.67	4.94	227.00
**Apo-B**	$1.62 \cdot 10^4$	$8.44 \cdot 10^3$	0.99	1.91	859.34	5.72	2578.03
**Apo-C1**	$2.69 \cdot 10^3$	$-4.43 \cdot 10^4$	0.99	4.04	31.11	12.11	93.32
**Apo-C2**	$6.02 \cdot 10^4$	$5.98 \cdot 10^5$	0.99	1.73	18.81	5.20	56.42
**Apo-C3**	$5.00 \cdot 10^3$	$-1.19 \cdot 10^5$	0.99	20.03	180.87	60.08	542.60
**Apo-C4**	$3.65 \cdot 10^4$	$-7.26 \cdot 10^4$	0.99	1.80	24.26	5.39	72.77
**Apo-D**	$4.03 \cdot 10^4$	$2.40 \cdot 10^5$	0.99	2.06	35.46	6.18	106.38
**Apo-E**	$1.30 \cdot 10^4$	$-1.47 \cdot 10^5$	0.99	5.01	120.51	15.03	361.54

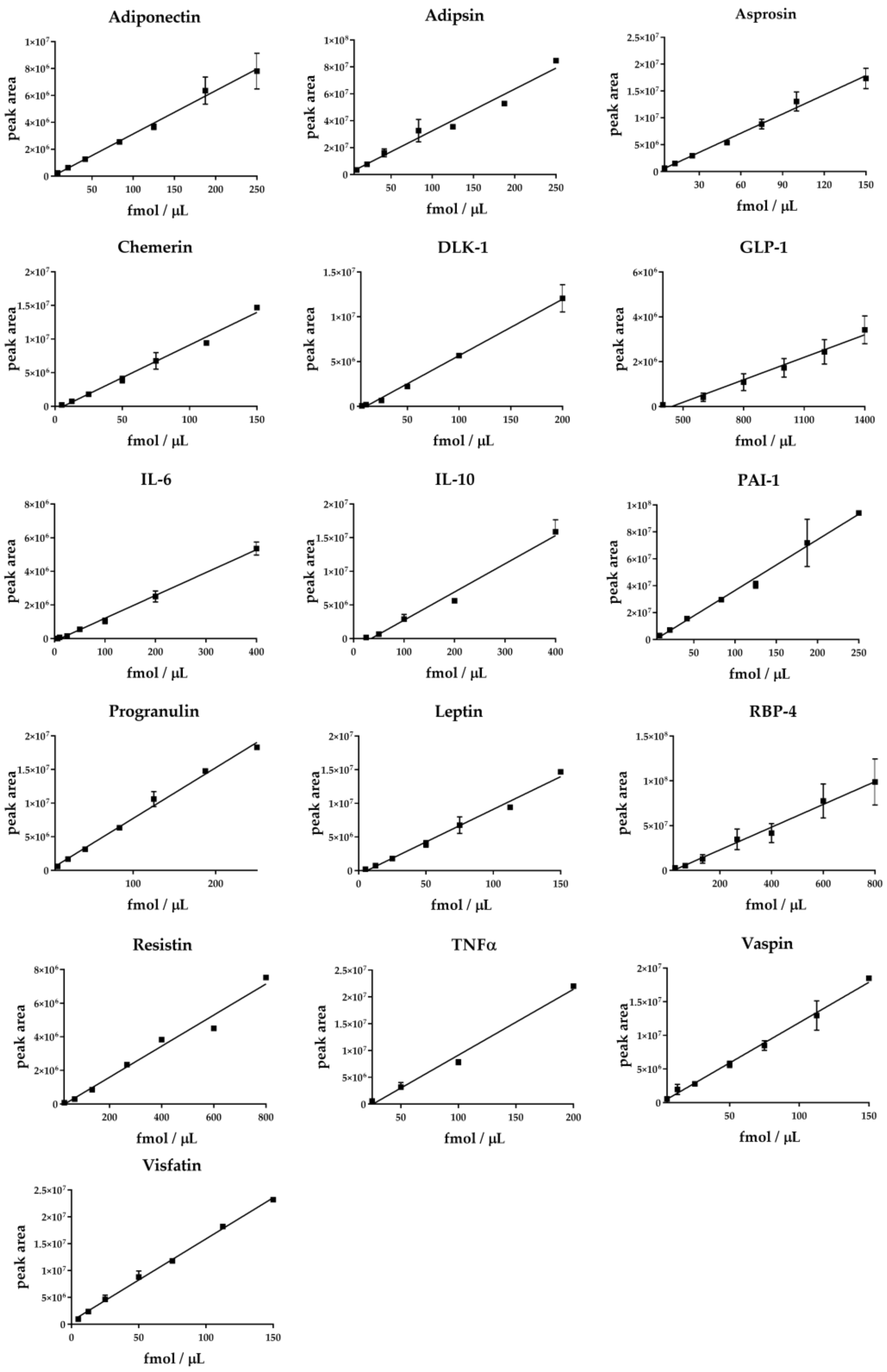

Figure S1. Calibration curves (n=5) of 16 adipokines. Isotope-labeled peptides were spiked in to HSA/IgG-depleted human serum and serially diluted.

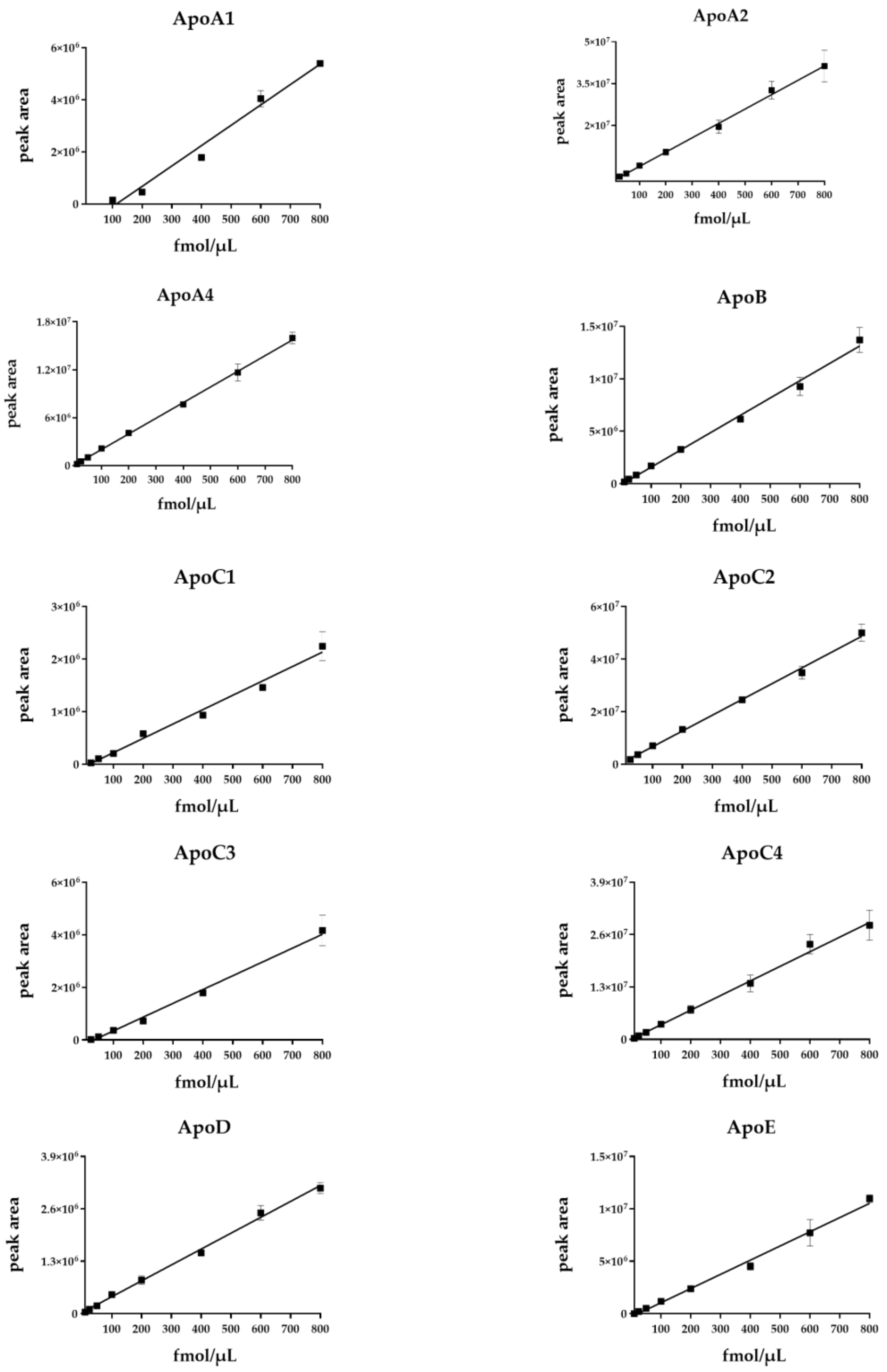

Figure S2. Calibration curves (n=5) of 10 apolipoprotein. Isotope-labeled peptides were spiked into HSA/IgG-depleted human serum and serially diluted.
